# Influence of Inspiratory Muscle Training on Ventilatory Efficiency and Cycling Performance in Normoxia and Hypoxia

**DOI:** 10.3389/fphys.2017.00133

**Published:** 2017-03-08

**Authors:** Eduardo Salazar-Martínez, Hannes Gatterer, Martin Burtscher, José Naranjo Orellana, Alfredo Santalla

**Affiliations:** ^1^Department of Sports and Computing, Pablo de Olavide UniversitySeville, Spain; ^2^Department of Sport Science, Medical Section, University of InnsbruckInnsbruck, Austria

**Keywords:** *V*_E_/VCO_2_ slope, cycling performance, ventilation, chemosensitivity, time trial

## Abstract

The aim of this study was to analyse the influence of inspiratory muscle training (IMT) on ventilatory efficiency, in normoxia and hypoxia, and to investigate the relationship between ventilatory efficiency and cycling performance. Sixteen sport students (23.05 ± 4.7 years; 175.11 ± 7.1 cm; 67.0 ± 19.4 kg; 46.4 ± 8.7 ml·kg^−1^·min^−1^) were randomly assigned to an inspiratory muscle training group (IMTG) and a control group (CG). The IMTG performed two training sessions/day [30 inspiratory breaths, 50% peak inspiratory pressure (Pimax), 5 days/week, 6-weeks]. Before and after the training period subjects carried out an incremental exercise test to exhaustion with gas analysis, lung function testing, and a cycling time trial test in hypoxia and normoxia. Simulated hypoxia (FiO_2_ = 16.45%), significantly altered the ventilatory efficiency response in all subjects (*p* < 0.05). Pimax increased significantly in the IMTG whereas no changes occurred in the CG (time × group, *p* < 0.05). Within group analyses showed that the IMTG improved ventilatory efficiency (*V*_E_/VCO_2_ slope; EqCO_2_VT_2_) in hypoxia (*p* < 0.05) and cycling time trial performance [W_TTmax (W)_; W_TTmean (W)_; PTF_(W)_] (*p* < 0.05) in hypoxia and normoxia. Significant correlations were not found in hypoxia nor normoxia found between ventilatory efficiency parameters (*V*_E_/VCO_2_ slope; LEqCO_2_; EqCO_2_VT_2_) and time trial performance. On the contrary the oxygen uptake efficiency slope (OUES) was highly correlated with cycling time trial performance (*r* = 0.89; *r* = 0.82; *p* < 0.001) under both conditions. Even though no interaction effect was found, the within group analysis may suggest that IMT reduces the negative effects of hypoxia on ventilatory efficiency. In addition, the data suggest that OUES plays an important role in submaximal cycling performance.

## Introduction

Ventilatory efficiency can be defined as the relationship between carbon dioxide production (VCO_2_) and ventilation (*V*_E_). Increased *V*_E_ and the removal of CO_2_ during physical exercise are essential for homeostatic control of whole body pH (Brown et al., 2013). There are four common ways for measuring ventilatory efficiency during an incremental test: (a) using the slope of the relationship between VCO_2_ and *V*_E_ (*V*_E_/VCO_2_ slope; Ingle et al., 2007), (b) the lowest equivalent of CO_2_during the incremental test (LEqCO_2_; Sun et al., 2002), (c) the equivalent of CO_2_ at the second ventilatory threshold (EqCO_2_VT_2_; Sun et al., 2002), and (d) the oxygen uptake efficiency slope (OUES; Baba et al., 1999a). Generally, a lower equivalent of CO_2_ indicates a greater ventilatory efficiency (Sun et al., 2002). The OUES represents the rate of increase of VO_2_ in response to a given *V*_E_ during incremental exercise, indicating how effectively oxygen is extracted and taken into the body (Baba et al., 1996).

In the clinic field ventilatory efficiency has been widely used as a prognostic marker to determine exercise limitation (Ingle et al., 2007; Arena et al., 2008; Laveneziana et al., 2010). Indeed, a relationship has been reported between sudden dead risk in hypertrophic cardiomyopathy and ventilatory efficiency (Magrì et al., 2016). However, the importance of the ventilatory efficiency for sport performance remains unclear. On one hand, Brown et al. (2013) did not find a relationship between maximum oxygen uptake (VO_2max_) and OUES in juvenile cyclists. In the same way, we did not find a relationship between VO_2max_, peak power output (PPO), and *V*_E_/VCO_2_ slope in world-class cyclists (Salazar-Martínez et al., 2016). On the other hand, a significant correlation was found between OUES and VO_2max_ in young active women (Mourot et al., 2004).

In hypoxia, the reduced partial pressure of oxygen (PO_2_) and the resulting arterial desaturation stimulates *V*_E_ (Babcock et al., 1995). Although the increased V_E_ during exercise in hypoxia (FIO_2_ = 0.15) increases PaO_2_ (Warner and Mitchell, 1990) it also leads to a higher oxygen cost of breathing compared to normoxia (Babcock et al., 1995). Additionally, in hypoxia, *V*_E_ may increase in excess of what would be required to maintain partial pressure levels of carbon dioxide (PaCO_2_; Warner and Mitchell, 1990). Therefore, a certain ventilatory inefficiency could be expected in hypoxia due to this overshoot in *V*_E_. However, high ventilatory efficiency is essential to maintain adequate level of PaO_2_ and PaCO_2_ with a lower breathing work in high altitude (Bernardi et al., 2006). In this regard, it may be assumed that efficient breathing may play an important role in the regulation of PaO_2_ and PaCO_2_ and achieving a higher sport performance in hypoxia.

In accordance, inspiratory muscle training (IMT) has been shown to be an effective method to improve both the ventilatory response in normoxia and hypoxia (Downey et al., 2007; Esposito et al., 2010) and the alveolar-arterial gradient in hypoxia (Esposito et al., 2010). Thus, it could be speculated that well-trained inspiratory muscles may help to preserve PaO_2_ in hypoxia due to improved ventilation-perfusion matching and to prevent excessive CO_2_ output due to less hyperventilation. However, to the best of our knowledge, whether or not IMT may improve the ventilatory efficiency under hypoxia conditions has not yet been tested.

Next to the effect on ventilation, IMT has been shown to improve sport performance as well (Romer et al., 2002a; Wells et al., 2005). However, the mechanisms responsible for the performance improvements after IMT remain controversial (Edwards and Walker, 2009). The mechanisms suggested to improve performance include a hypertrophy of diaphragm (Downey et al., 2007), an increase in blood flow to the locomotor muscles (Harms et al., 1997) and a reduction in subjective perception of fatigue and dyspnea ratings (Downey et al., 2007). Additionally, Sheel (2002) hypothesized that changes in performance after IMT could be related to improvements on ventilatory efficiency. However, to the best of our knowledge there are no studies evaluating the relationship between changes in sport performance after IMT and ventilatory efficiency. After IMT, the metabolic demand of the inspiratory muscles during exercise are reduced (Babcock et al., 1995), thus contributing to a lower overall O_2_ uptake and CO_2_ output. In situations where the ventilatory efficiency is impaired, for example in hypoxia, such effects may influence exercise performance (Roussos, 1985).

Therefore, the aim of this study was (a) to evaluate the influence of IMT on ventilatory efficiency in normoxia and hypoxia, and (b) to investigate the relationship between ventilatory efficiency and cycling performance under both conditions.

We hypothesized that IMT improves ventilatory efficiency in normoxia and especially in hypoxia and reduces the metabolic demands of the respiratory muscles in both conditions. We also hypothesized that improvements in submaximal cycling performance can be linked to improvements in ventilatory efficiency in normoxia and hypoxia.

## Materials and methods

### Subjects

Sixteen physically active and healthy participants [*n* = 9 male (23.44 ± 2.7 years; 180.22 ± 3.5 cm; 78.2 ± 5.5 kg; 48.39 ± 7.28 ml·kg^−1^·min^−1^); *n* = 7 female (25.37 ± 3.24 years; 168.75 ± 5.1 cm; 62.62.2 ± 9.47 kg; 38.15 ± 6.57 ml·kg^−1^·min^−1^)] were selected for the study. Each participant completed a health questionnaire before being included in the study. Participants with health diseases, breathing problems, or obstructive defects were excluded from the study. Before starting the study, written informed consent was obtained from each participant in accordance with the Declaration of Helsinki. The study was approved by the Ethics Committee of the University of Innsbruck.

### Design

Participants were randomly assigned to either an inspiratory muscle training group (IMTG) or a control group (CG). The IMTG performed two training sessions per day, 5 days per week during a period of 6-weeks. Each participant completed 30 inspiratory breaths with a PowerBreath device (PowerBreathe®, K3) at 50% of their individual Pimax. Inspiratory training load was adjusted weekly at 50% of the individual Pimax. Every training session was performed under expert supervision. The CG did not carry out any inspiratory training during the experimental period. This procedure seems to be adequate as a placebo effect is not expected. For instance, when considering differences between trials that included a control group and studies that did not, 69% of the placebo-controlled studies showed a positive outcome for RMT (i.e., performance improvements for the RMT groups significantly exceeded those for the control groups), which is very similar to the 75% positive outcomes of the studies without any controls (Illi et al., 2012). Participants were advised not to change normal physical training habits during the experimental period.

### Pulmonary function tests

Before and after the experimental period, participants performed lung function testing (Schiller SP-1®, Switzerland) to assess the forced vital capacity (FVC), forced expiratory volume during the first second (FEV_1_), the ratio between forced expiratory capacity during the first second and vital capacity (FEV_1_/VC), the peak expiratory flow (PEF), and the peak inspiratory flow (PIF; Table 1). The best attempt out of three tests was included in the analysis. Peak inspiratory mouth pressure (Pimax) was determined with a portable device (PowerBreathe®, K3). During the Pimax test participants had to inspire as fast as possible from a normal expiration. Each participant repeated the test until the measurements were stable. Pimax was measured weekly using the same testing protocol.

**Table 1:** **Results of pulmonary function testing pre and post experimental period (Mean ± ***SD***)**.

	**IMTG**		**CG**	
	**Pre**	**Post**	**Pre**	**Post**
FVC (l)	5.44 ± 1.14	4.67 ± 1.38	5.06 ± 1.17	4.96 ± 0.93
FEV_1_ (l)	4.64 ± 0.92	4.19 ± 0.8	4.31 ± 0.85	4.06 ± 0.79
FEV_1_/VC (%)	84.13 ± 11.58	82.51 ± 9.19	82.33 ± 6.28	79.84 ± 6.48
PEF (l·s^−1^)	9.27 ± 2.23	8.2 ± 1.53	8.9 ± 2.47	8.73 ± 2.4
PIF (l·s^−1^)	7.04 ± 1.92	8.31 ± 2.39	7.12 ± 1.2	7.57 ± 2.2
Pimax (cm H_2_O)	119.6 ± 37.36	166.91 ± 42.65^*^	130.55 ± 33.58	146.72 ± 40.62

*FVC, forced vital capacity; FEV1, forced expiratory volume during the first second; FEV1/VC, ratio between forced expiratory volume during the first second and vital capacity; PEF, peak expiratory flow; PIF, peak inspiratory flow; Pimax, peak inspiratory pressure*.

* *p < 0.05 post vs. pre training*.

### Incremental exercise testing

Before (Pre) and after (Post) the training period participants performed maximum incremental exercise tests in normoxia and hypoxia (overall four tests). Each test was separated by 48 h. During the tests, oxygen uptake (VO_2_), carbon dioxide output (VCO_2_), respiratory exchange ratio (RER), ventilation (*V*_E_), breathing frequency (BF), tidal volume (VT), oxygen equivalent (EqVO_2_), and carbon dioxide equivalent (EqCO_2_) were measured breath by breath with a portable gas analyser (Jaeger Oxygen™®, Germany). The system was calibrated prior to each test with gas mixtures of known concentration. Tests were carried out on a cycle ergometer (RBM Cyclus 2®, Germany). After 4 min of warming up, participants started the test at 50 W and then the load was increased by 25 W each minute until volitional exhaustion. Achievement of maximum oxygen uptake (VO_2max_) was accepted when a plateau was found in the relationship between VO_2_ and power output or when three of the four criteria for maximal VO_2max_ were obtained (Howley et al., 1995). Tests were carried out at approximately the same time of the day in an air-conditioned normobaric hypoxic chamber (size 4.75 × 2.25 m, LowOxygen®, Germany). During the normoxia testing the hypoxic chamber was switched off whereas during the hypoxia setting the chamber was set at a simulated altitude of 2,500 m (FiO_2_ = 16.45%). Participants were blinded to the simulated altitude of the hypoxic chamber. Participants were advised to avoid exhausting exercise 1 day before the tests and to take any ergogenic aids (e.g., caffeine).

### Time trial performance

Ninety minutes after the incremental test, cycling endurance performance was evaluated by a 10 min time trial (TT). The cycle ergometer was shifted to a fixed pedal force in which power output was dependent on the pedaling rate. Pedal force for each participant was set in order that pedaling at 90 rpm produced 85% (rounded to 5 W) of peak power output determined by the incremental cycle ergometer test. During the test, cyclists were strongly encouraged to choose a maximal pedaling rate that could be maintained for the respective test duration. As with the incremental test, each participant performed the TT, under normoxic (TT_nor_) and under hypoxic conditions (TT_hyp_) separated by 48 h, before and after the experimental period. During each TT-test peak power output (W_max_), mean power output (W_mean_), and pedal torque force (PTF) were recorded.

### Test–retest reproducibly of time trial test

**Figure 1:**
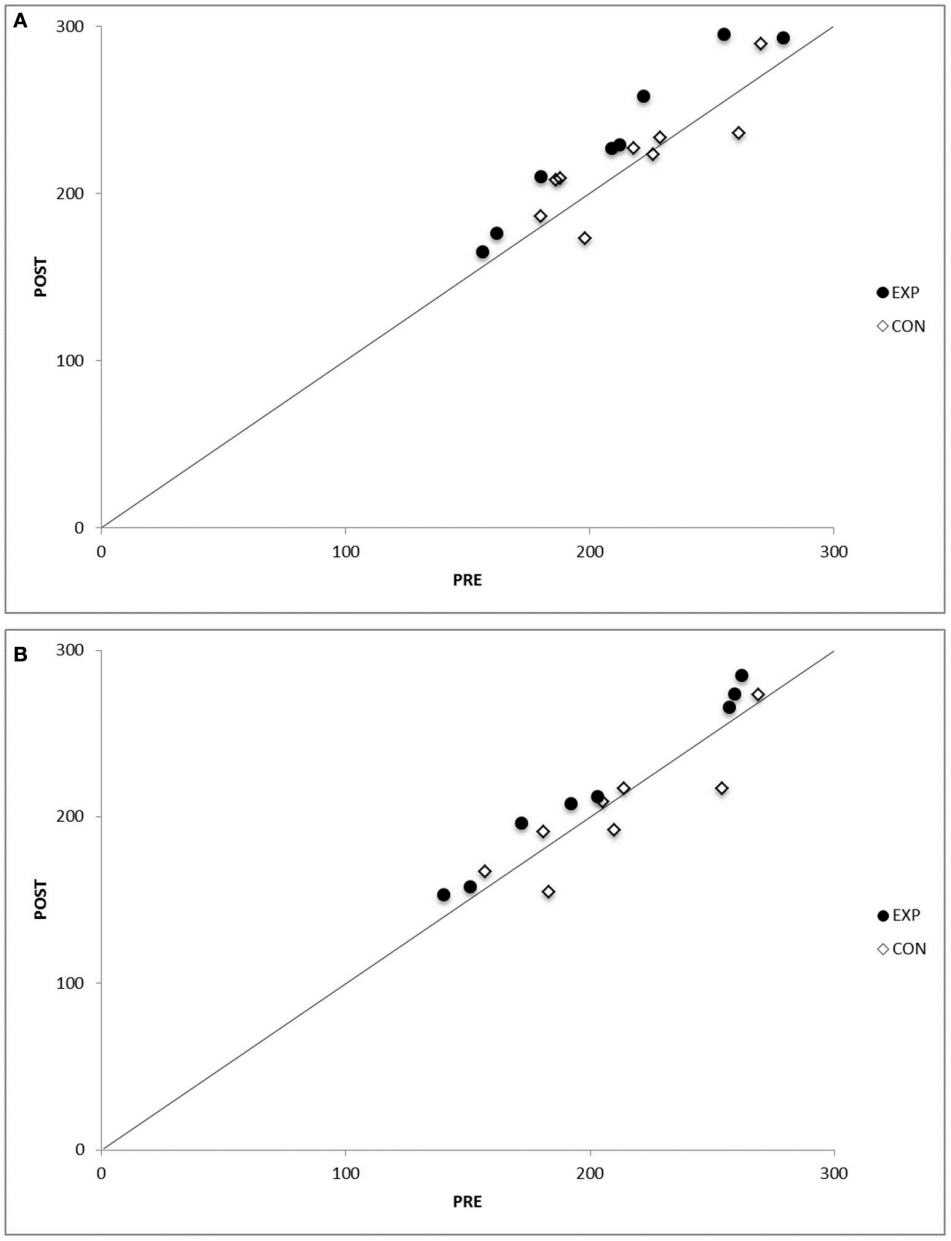
**(A)** Test–retest reproducibility in the control/experimental group subjects during the time trial test (TT) test, before (Pre), and after (Post) the intervention period in normoxia. Identity lines are drawn in both graphs. See text for numerical analysis. **(B)** Test–retest reproducibility in the control/experimental group subjects during the time trial test (TT) test, before (Pre), and after (Post) the intervention period in hypoxia. Identity lines are drawn in both graphs. See text for numerical analysis.

The coefficient of variation for the time trial test in the control group in normoxia was 14.9% in the pre-test and 15.9% in the post-test (Figure 1A). In hypoxia, the coefficient of variation for the time trial test in the control group was 17.9% in the pre-test and 17.8% in the post-test (Figure 1B). The intra-class correlation coefficient for the time trial test in the control group was 0.92 in normoxia and 0.93 in hypoxia.

### Ventilatory efficiency

The *V*_E_/VCO_2_ slope was calculated from the slope of the relationship between VCO_2_ and *V*_E_ during each incremental exercise test. To exclude the influence of the respiratory compensation due to acidosis during highly intensive exercise, the *V*_E_/VCO_2_ slope was determined from the beginning of the test until the second ventilatory threshold (VT_2_). VT_2_ was identified by an increase in the ventilatory equivalent of CO_2_ (EqCO_2_) and a decrease in end tidal partial pressure of carbon dioxide (PETCO_2_; Lucía et al., 2000). Oxygen uptake efficiency slope (OUES) was calculated from the linear relationship of VO_2_ vs. the logarithm of *V*_E_ during exercise (VO_2_ = a log_10_ *V*_E_ + b).

### Statistics analysis

Data are expressed as mean ± *SD* for each variable. The statistical power for the chosen sample size of 16 participants (9 in the IMTG and 7 in the CG) was >90%; alpha = 0.05. The power calculation (G^*^Power 3.1.7) was based on expected changes in Pimax and TT performance (W_TTmean_) due to IMT (Romer et al., 2002a). The normal distribution of the data was checked by the Shapiro-Wilk test. The homogeneity of variance was evaluated by Levene's test. To compare the values obtained for each variable during the test, mixed-effects ANOVA test was used (group × time × condition). When significant differences were found, the Bonferroni test was used as a *post-hoc* test. ANOVA test was also applied to evaluate a possible gender effect (group × time × condition × gender). Effect size (ES) was calculated when a significant difference was found. A correlation analysis (Pearson-coefficient) was carried out between TT performance variables, incremental test variables and ventilatory efficiency variables with data from both groups and from both test in two different situations (normoxia and hypoxia; **Table 6**). Linear regression analysis was performed between Pimax, *V*_E_/VCO_2_ slope and OUES (dependent variables) and TT performance (independent variable) with data from both groups (IMTG and CG) and from both tests (Pre and Post) in normoxia. The level of significance was set at *p* < 0.05 for each statistical analysis.

## Results

No gender effect was identified with regard to the parameters of interest (*V*_E_/VCO_2_ slope, LEqCO_2_, EqCO_2_VT_2_, OUES). Baseline values did not differ between groups (*V*_E_/VCO_2_ slope, LEqCO_2_, EqCO_2_VT_2_, OUES, Pimax, PPO, TT_Wmean_). Outcomes of the pulmonary function testing before and after the experimental period are shown in Table 1. Significant differences were found in Pimax between Pre- and Post-test in the IMTG (*p* < 0.05).

### Inspiratory muscle training (IMT)

**Figure 2:**
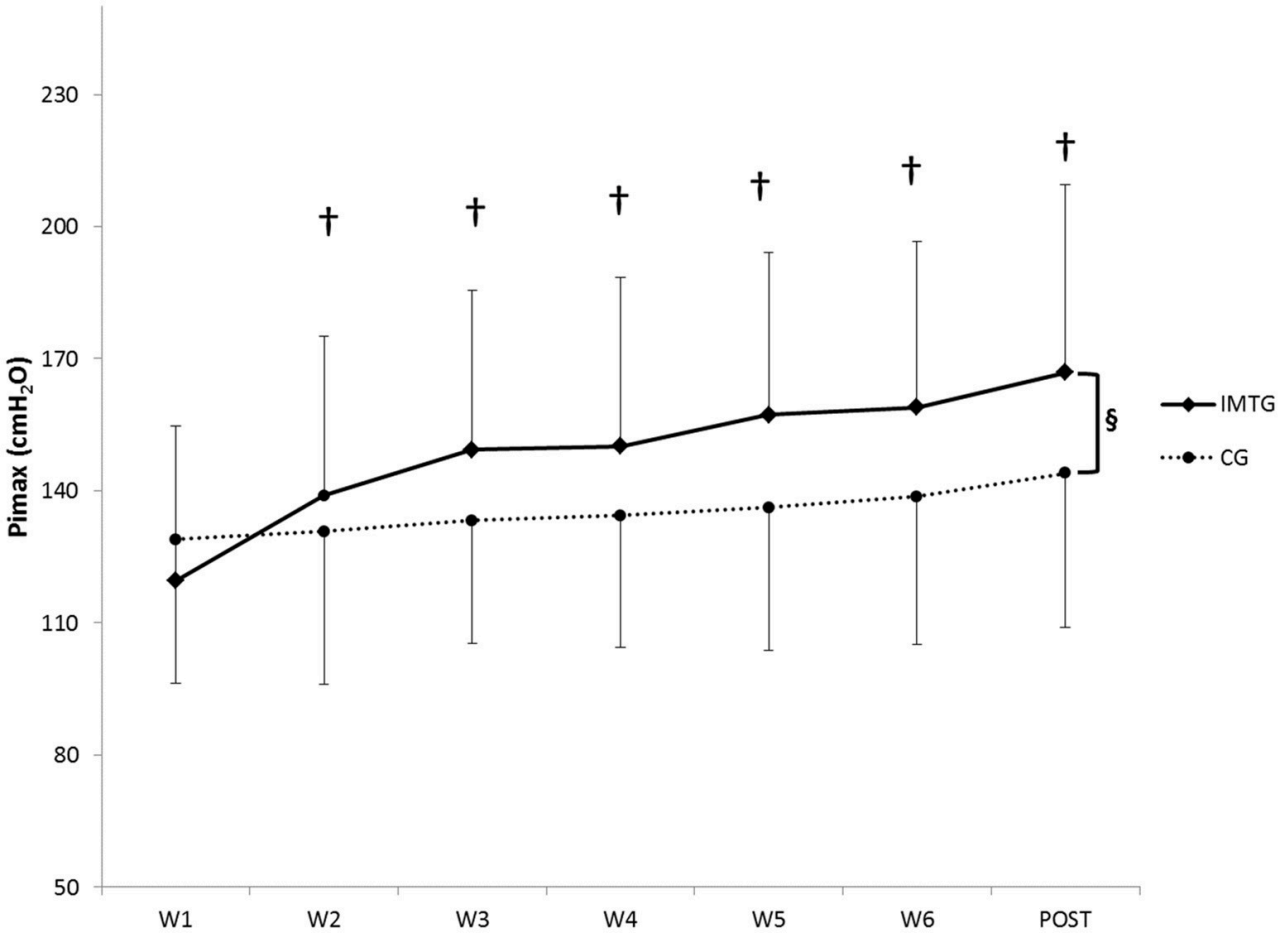
**Weekly values of Pimax (Mean ± ***SD***) for the inspiratory muscle training group (IMTG) and control group (CG)**. ^§^Two-way ANOVA for repeated measures (time × group) interaction (*p* < 0.05). ^†^Differences from baseline evaluation (Bonferroni test).

Figure 2 shows the changes in Pimax during the experimental period in both groups. Mean Pimax improved significantly from week-1 to week-6 in the IMTG (+28.37%, *p* < 0.05) with no improvements in the CG (interaction effect, time × group, *p* < 0.05).

### Ventilatory efficiency

The group comparison of the ventilatory efficiency variables are shown in **Table 3**. There were no group differences and no interaction effect in any of the established variables at Pre and Post in normoxia and hypoxia. Significant differences were found between normoxia and hypoxia in the *V*_E_/VCO_2_ slope, LEqCO_2_, and EqCO_2_VT_2_ in the Pre-test in both groups (*p* < 0.05; Table 2). During the Post-test significant differences between normoxia and hypoxia were found in LEqCO_2_ and OUES in the IMTG and in *V*_E_/VCO_2_ slope, LEqCO_2_ and OUES in the CG (*p* < 0.05). In both groups significant differences in *V*_E_/VCO_2_ slope and EqCO_2_VT_2_ were found between Pre and Post in hypoxia (*p* < 0.05).

**Table 2:** **Evaluation of ventilatory efficiency variables in normoxia and hypoxia before the experimental period with data from both groups (Mean ± ***SD***)**.

	***V*_E_/VCO_2_ slope**	**LEqCO_2_**	**EqCO_2_VT_2_**	**OUES**
Normoxia (*n* = 16)	24.58 ± 2.95	22.63 ± 2.68	23.91 ± 2.34	3.24 ± 0.62
Hypoxia (*n* = 16)	29.15 ± 3.26^*^	24.8 ± 1.9^*^	27.28 ± 2.79^*^	2.96 ± 0.85^*^
% ΔChange	+18.5%	+9.58%	+14.09%	−8.64%

*V_E_/VCO_2_ slope, Slope of the relationship between VCO_2_ and V_E_; LEqCO_2_, lowest equivalent of CO_2_ during the incremental test; EqCO_2_VT_2_, equivalent of CO_2_ in the second ventilatory threshold; OUES, oxygen uptake efficiency slope; % ΔChange, Percentage of change between measurements*.

* *t-Test for paired samples (p < 0.05)*.

### Time trial performance

Time trial performance parameters are shown in **Table 4**. During Pre- and Post-test, significant differences between normoxia and hypoxia were found in W_TTmean (W)_ and in W_TTmean (W/Kg)_ in both groups (*p* < 0.05). There was no interaction effect in these variables. However, after the experimental period, W_TTmean (W)_ and W_TTmean (W/Kg)_ were significantly higher in normoxia and hypoxia in the IMTG (*p* < 0.05). At post, significant differences were found in W_max_ between normoxia and hypoxia in both groups (*p* < 0.05). A significant reduction in PTF was found in the CG in hypoxia in both tests (Pre and Post) and in the Post-test in the IMTG (*p* < 0.05). A significant increase in PTF was found in the IMTG in normoxia after IMT (*p* < 0.05).

### Incremental exercise testing

VO_2max_ was reduced in hypoxia in both groups compared to normoxia in the Post-test (*p* < 0.05). Compared to normoxia, PPO was reduced in both groups during the Pre- and Post-test in hypoxia (*p* < 0.05). There was no interaction effect. However, PPO increased significantly in the IMTG in normoxia after the experimental period compared to the Pre-test evaluation (*p* < 0.05). Before the experimental period, *V*_Emax_ increased in hypoxic conditions in both groups. After the experimental period, *V*_Emax_ increased only in the CG. However, all these variations were not significant in both gorups. VT_max_ and BF_max_ did not change in any condition.

### Correlation and regression analysis

**Figure 3:**
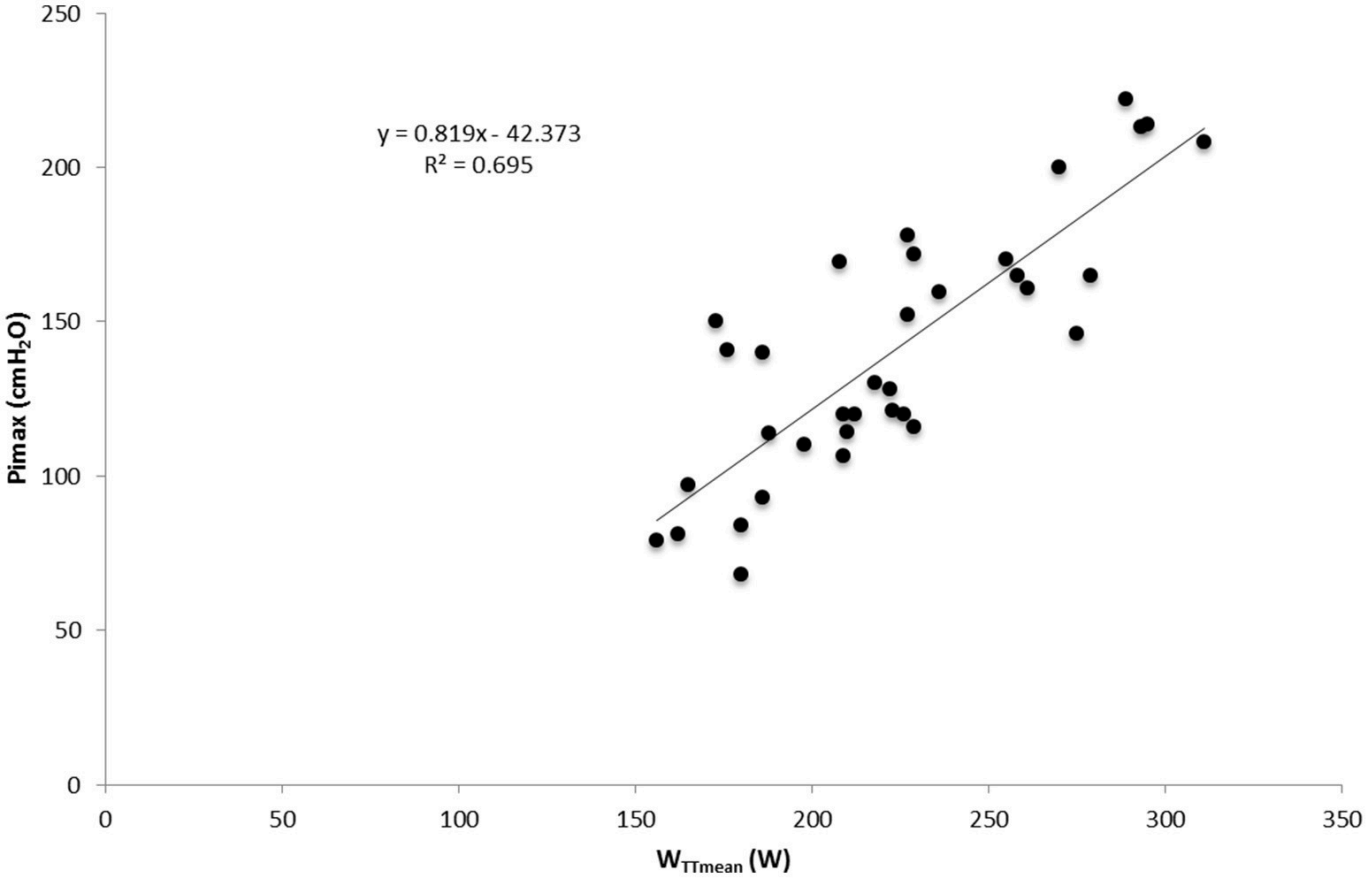
**Relationship between inspiratory muscle strength (Pimax) and cycling time trial (TT) performance with data from both groups and both test in normoxia**.

**Figure 4:**
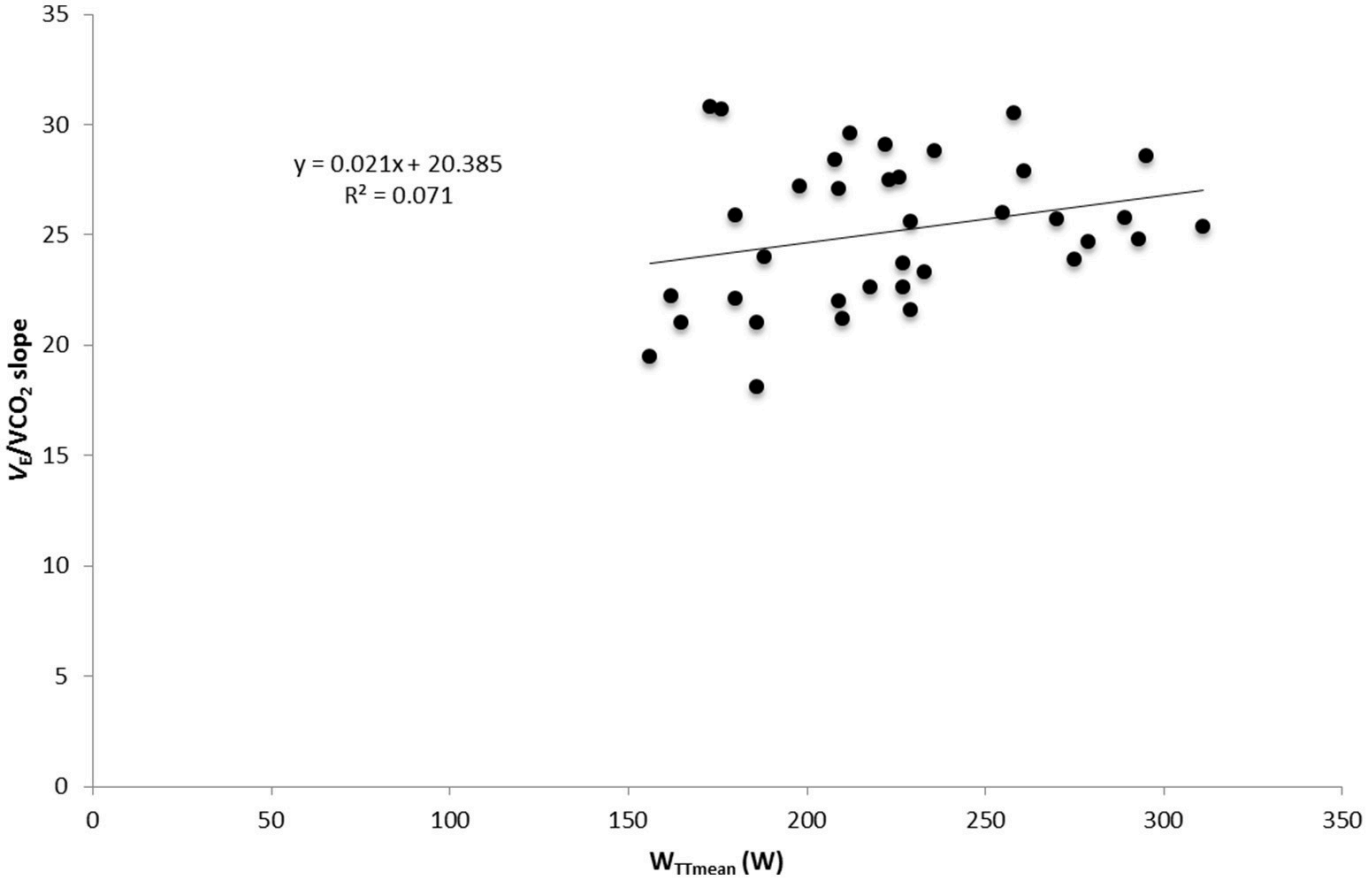
**Relationship between cycling time trial (TT) performance and ventilatory efficiency measured as ***V***_**E**_/VCO_**2**_ slope with data from both groups and both test in normoxia**.

**Figure 5:**
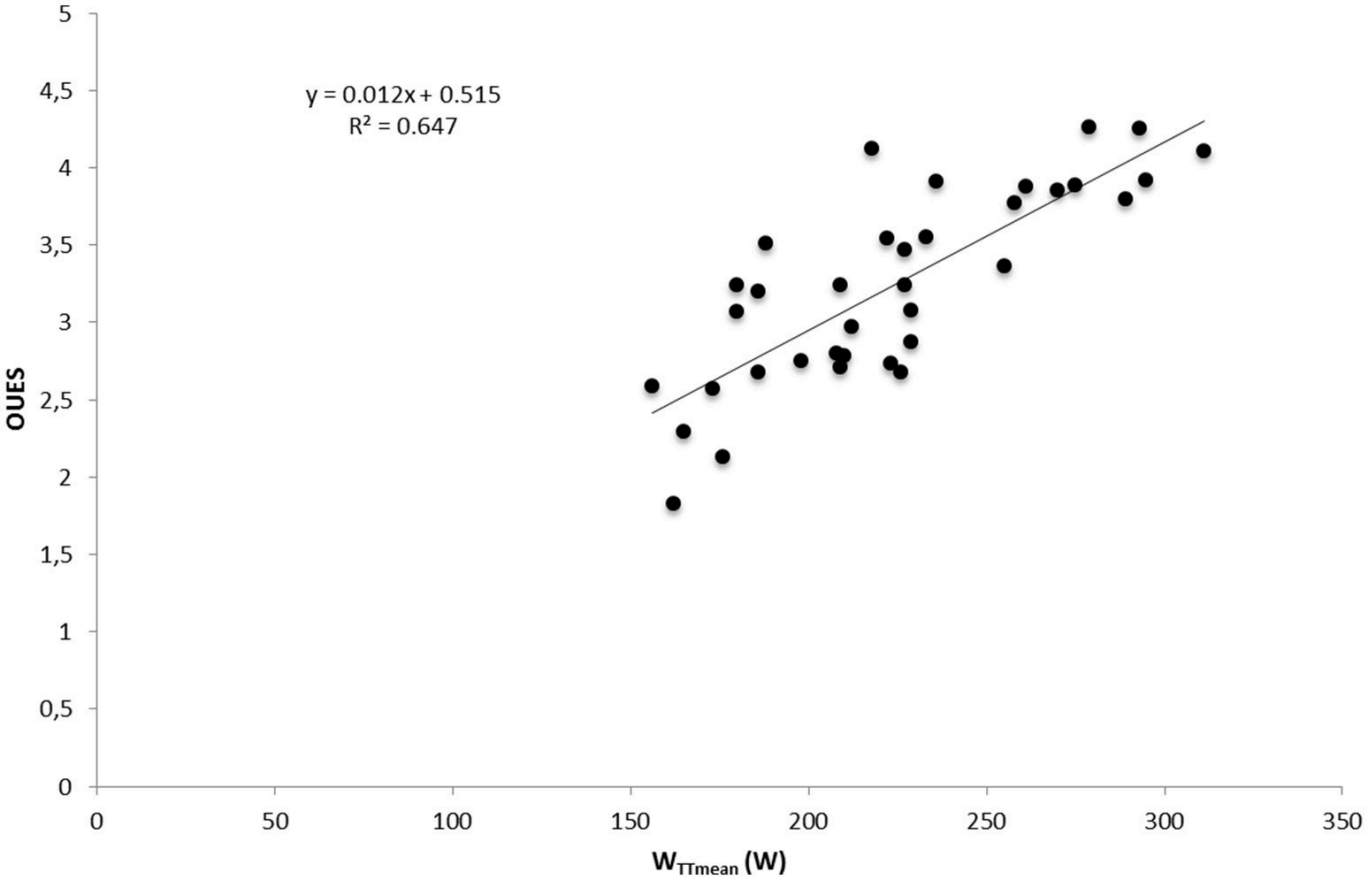
**Relationship between cycling time trial (TT) performance and ventilatory efficiency measured as OUES with data from both groups and both test in normoxia**.

Significant correlations were found between Pimax and W_TTmean_, VO_2max_, *V*_Emax_, and PPO with data from both test and both groups in normoxia (*p* < 0.05; **Table 6**). No correlation was found between ventilatory efficiency variables and performance variables. A significant correlation was found between OUES and maximal performance variables (VO_2max_, *V*_Emax_, PPO) and W_TTmean_ (*p* < 0.05) in normoxia and hypoxia (**Table 6**). A linear relationship was found between muscle breathing strength (Pimax) and TT performance in normoxia (*R*^2^ = 0.69, *p* = 0.00; Figure 3) and in hypoxia (*R*^2^ = 0.67, *p* = 0.00). No relationship was found between time trial performance (W_TTmean_) and ventilatory efficiency (*V*_E_/VCO_2_ slope) in normoxia (*R*^2^ = 0.149, *p* = 0.02; Figure 4) and in hypoxia (*R*^2^ = 0.02, *p* = 0.81). W_TTmean_ and OUES were significantly related in normoxia (*R*^2^ = 0.647, *p* = 0.00; Figure 5) and in hypoxia (*R*^2^ = 0.631, *p* = 0.01).

## Discussion

To the best of our knowledge, this is the first study that investigated the effects of IMT on ventilatory efficiency variables in normoxic and hypoxic conditions. We hypothesized that IMT could improve the ventilatory efficiency response in normoxia and hypoxia. We also hypothesized that improvements in the submaximal cycling performance may be linked to improvements in ventilatory efficiency. The main finding of this study was that IMT improved *V*_E_/VCO_2_ slope (−7.95%) in hypoxia and TT performance in both normoxia (+10.17%) and hypoxia (+6.62%) conditions. However, despite this within-group effect no interaction effect was found. Additionally, cycling TT performance was positively related to the OUES in normoxia and hypoxia and to the inspiratory muscle strength (Pimax). These findings partly support (only in OUES) the hypothesis that changes in sport performance after IMT may be linked to changes in ventilatory efficiency.

It is well known that *V*_E_ is stimulated in hypoxia mediated by stimulation of the peripheral chemoreceptors (Dempsey and Forster, 1982; Townsend et al., 2002). However, the influence of hypoxic conditions on ventilatory efficiency has not been thoroughly investigated. In the present study, hypoxia at Pre worsened the ventilatory efficiency response in both groups (+18.5% *V*_E_/VCO_2_ slope; +9.58% LEqCO_2_; +14.09% EqCO_2_VT_2_; and −8.64% OUES, respectively; Table 2), which is in agreement with the increase in the ventilatory equivalents described at 16% FiO_2_ (Ozcelik and Kelestimur, 2004). The increased *V*_E_ at altitude, initiated to maintain SaO_2_ (Rusko et al., 2004; Burtscher et al., 2006; Faiss et al., 2014), may to some extent explain the deterioration of ventilatory efficiency. In addition, in hypoxia *V*_E_ may be increased in excess of what would be required to maintain partial pressure levels of carbon dioxide (PaCO_2_; Warner and Mitchell, 1990) thus influencing the relationship between *V*_E_ and VCO_2_.

After the training period, hypoxia did not significantly increase the V_E_/VCO_2_ slope response in the subjects that performed the IMT [+3.43% (ES = 0.4) vs. +9.48% (ES = 1.3) for the IMTG and CG, respectively]. These changes might be explained by improvements in breathing muscle strength (Pimax) and altered breathing patterns after IMT. Respiratory muscle training has been shown as an effective method to improve A-a gradient and ventilation–perfusion mismatch (Esposito et al., 2010). A better A-a gradient in hypoxia might have reduced the V_E_ overshoot observed in hypoxia before training (Table 3). In support of this, it has been reported that climbers who managed to climb Mt. Everest and K2 without oxygen, are those with a high ventilatory efficiency and “optimized” breathing patterns (Bernardi et al., 2006). Again, it has to be underlined that no interaction effect existed with respect to the altered V_E_/VCO_2_ slope in hypoxia after the training period. Therefore, the reported training effect contains some uncertainty and further studies with a greater sample size are needed to confirm our conclusions.

**Table 3:** **Comparison between groups in ventilatory efficiency variables in the four experimental conditions**.

**IMTG**	**Pre**		**Post**		**ANOVA**			
	**Normoxia**	**Hypoxia**	**Normoxia**	**Hypoxia**	**Main effect (time)**	**Main effect (condition)**	**Main effect (group)**	**Interaction (condition × group × time)**
*V*_E_/VCO_2_ slope	23.68 ± 2.94	28.77 ± 2.74# (1.3)	25.6 ± 3.95	26.48 ± 2.77^*^ (0.42)	0.267	0.000	0.476	0.313
LEqCO_2_	22.51 ± 1.32	24.72 ± 1.45# (1.2)	22.73 ± 1.65	23.98 ± 1.64# (0.83)	0.609	0.000	0.755	0.519
EqCO_2_VT_2_	23.68 ± 2.33	27.26 ± 2.94# (1.3)	24.32 ± 2.92	24.38 ± 2.11^*^ (1.5)	0.022	0.000	0.733	0.233
OUES	3.22 ± 0.75	3.13 ± 0.82	3.31 ± 0.83	2.92 ± 0.71# (0.81)	0.493	0.007	0.994	0.203
**CG**								
*V*_E_/VCO_2_ slope	25.31 ± 2.35	29.81 ± 3.7# (1.7)	25.63 ± 3.93	28.06 ± 3.5^*^# (1.2^*^−1.3#)	–	–	–	–
LEqCO_2_	22.75 ± 3.59	24.87 ± 2.17# (0.5)	22.62 ± 1.98	24.73 ± 1.58# (1.9)	–	–	–	–
EqCO_2_VT_2_	24.26 ± 2.05	27.15 ± 2.81# (0.8)	24.15 ± 3.11	25.7 ± 2.73^*^ (0.8)	–	–	–	–
OUES	3.36 ± 0.56	2.98 ± 0.91	3.24 ± 0.52	3.01 ± 0.55# (1.0)	–	–	–	–

Data are presented as Mean ± SD and Effect size (ES). ES is showed when a statistical difference was found. V_E_/VCO_2_ slope, Slope of the relationship between VCO_2_ and V_E_; LEqCO_2_, lowest equivalent of CO_2_ during the incremental test; EqCO_2_VT_2_, equivalent of CO_2_ in the second ventilatory threshold; OUES, oxygen uptake efficiency slope. ANOVA mixed-effects Bonferroni post-hoc test:

* *Mixed-effects ANOVA Pre vs. Post in the same condition (p < 0.05)*.

# *Mixed-effects ANOVA Nor vs. Hyp at the same time (p < 0.05)*.

With regard to cycling performance, TT performance was reduced significantly in both groups before IMT in hypoxia (Table 4). After IMT, only the IMTG improved TT performance in normoxia and hypoxia (Table 4). Our results support previous studies showing a positive effect of IMT on sport performance (Volianitis et al., 2001; Romer et al., 2002a,b; Edwards and Walker, 2009). However, the participants of the present study not only improved their performance in normoxia (+11.33%), they also improved their performance in hypoxia (+7.33%) despite a reduction in VO_2max_ (−5.42%; Table 4). It could be hypothesized that IMT reduced the oxygen cost of the breathing muscles allowing higher O_2_ availability for the locomotor muscles. In addition, it has been suggested that reductions in respiratory effort could lead to greater locomotor muscle recruitment mediated by central nervous system control (Edwards and Walker, 2009). Once more, it should be noted that despite the improvements found in the IMTG no interaction effect was found. Thus, outcomes should be interpreted with some caution.

**Table 4:** **Comparison between groups in time trial variables in the four experimental conditions**.

**IMTG**	**Pre**		**Post**		**ANOVA**			
	**Normoxia**	**Hypoxia**	**Normoxia**	**Hypoxia**	**Main effect (time)**	**Main effect (condition)**	**Main effect (group)**	**Interaction (condition × group × time)**
W_TTmean_ (W)	217.25 ± 49.07	204.5 ± 49.67# (1.0)	241.87 ± 56.01^*^(1.9)	219 ± 51.22#^*^ (0.6#−0.6^*^)	0.026	0.000	0.755	0.611
W_TTmean_ (W/Kg)	3.03 ± 0.4	2.83 ± 0.45# (1.3)	3.35 ± 0.4^*^(2.4)	3.07 ± 0.44#^*^ (1.8#−1.5^*^)	0.041	0.000	0.823	0.610
W_TTmax_ (W)	296.25 ± 109.6	282.3 ± 112	319.12 ± 118	289 ± 105.9# (1.8)	0.969	0.000	0.353	0.769
PTF (W)	147.5 ± 22.83	141.25 ± 27.35	156.87 ± 29.51^*^ (1.1)	144.37 ± 27.57# (2.3)	0.466	0.000	0.676	0.164
**CG**								
W_TTmean_ (W)	221.25 ± 32.5	209.12 ± 37.47# (1.6)	222 ± 35.25	202.62 ± 36.23# (2.9)	–	–	–	–
W_TTmean_ (W/Kg)	3.08 ± 0.39	2.88 ± 0.43# (1.6)	3.11 ± 0.32	2.89 ± 0.32# (2.8)	–	–	–	–
W_TTmax_ (W)	273.28 ± 28.87	258.37 ± 36.89	265 ± 39.84	238.7 ± 37.45# (2.6)	–	–	–	–
PTF (W)	161.87 ± 22.19	146.87 ± 21.86# (1.7)	156.87 ± 22.98	144.75 ± 21.59# (1.0)	–	–	–	–

*Data are presented as Mean ± SD and Effect size (ES). ES is showed when a statistical difference was found. W_TTmax_, Peak power output; W_TTmean_ (W), mean watts; W_TTmean_ (W/Kg), mean watts per kilogram; PTF, pedal torque force. ANOVA mixed-effects Bonferroni post-hoc test*.

* *Mixed-effects ANOVA Pre vs. Post in the same condition (p < 0.05)*.

# *Mixed-effects ANOVA Nor vs. Hyp at the same time (p < 0.05)*.

A further finding of the present investigation was that most of the ventilatory efficiency variables (V_E_/VCO_2_ slope, LEqCO_2_ and EqCO_2_VT_2_) were not related to TT performance (**Table 6**; Figure 5). This is in contrast to the finding of Sheel (2002) who suggests that improvements in submaximal exercise performance after IMT are related to improvements in ventilatory efficiency. Nonetheless, results of the present investigation show a positive relationship between cycling time trial performance and respiratory muscle strength (r^2^ = 0.695; Figure 3). It could be argued that the increased respiratory muscle strength might reduce the oxygen cost of breathing during submaximal exercise, thus improving oxygen delivery to the working limb muscles. However, further studies are necessary to confirm this hypothesis. In contrast, OUES showed a linear relationship with cycling TT performance (r^2^ = 0.647; Figure 5). Subjects who showed a lower oxygen cost for the same increment in V_E_ are those who achieved a higher performance in the TT (W_TTmean_; **Table 6**). However, OUES was not modified by the IMT (Table 3) and was not related to Pimax (**Table 6**). Therefore, IMT seems to not play a role in this relationship. It should be mentioned that in contrast to our trained sample, OUES was modified by IMT in patients with heart failure and weakened breathing muscle (Winkelmann et al., 2009).

Regarding the incremental exercise test, VO_2max_ in hypoxia was reduced in both groups (−8.99% IMTG; −11.92% CG). Similar reductions were found previously at this simulated altitude (Lawler et al., 1988; Martin and O'kroy, 1993). Additionally, IMT did not improve VO_2max_ in normoxia and hypoxia (Table 5). Our results support previous studies that did not find an effect of IMT on VO_2max_ in normoxia and hypoxia (Downey et al., 2007; Esposito et al., 2010). However, the IMTG improved PPO after IMT in normoxia (+5.62%) and hypoxia (+2.51%) which is in contrast to previous studies that reported only a slight influence of IMT on PPO (Sheel, 2002; Illi et al., 2012). Moreover, except for OUES, the ventilatory efficiency variables were not correlated with performance variables of the incremental test (Table 6). Similar to the time trial outcome, OUES showed a strong correlation with VO_2max_ in normoxia and hypoxia (r = 0.89 and r = 0.82; respectively) and with PPO (r = 0.91 and r = 0.88; respectively). With respect to this finding, there are contrasting results reported in the literature. There are studies reporting a correlation between VO_2max_ and OUES (Baba et al., 1999a,b,c; Hollenberg and Tager, 2000) and others that did not find a correlation between these two parameters or only a weak correlation (Brown, 2010; Brown et al., 2013). Further research is necessary on the influence of IMT on ventilatory efficiency parameters in hypoxia.

**Table 5:** **Measured cardiorespiratory and performance variables at maximal exercise intensity in the four experimental conditions**.

**IMTG**	**Pre**		**Post**		**ANOVA**			
	**Normoxia**	**Hypoxia**	**Normoxia**	**Hypoxia**	**Main effect (time)**	**Main effect (condition)**	**Main effect (group)**	**Interaction (condition × group × time)**
VO_2max_ (ml·kg·min^−1^)	47.19 ± 9.45	45.15 ± 7.34	45.86 ± 5.07	43.37 ± 6.88#(0.6)	0.139	0.018	0.973	0.731
PPO (W)	289.37 ± 55.12	274.62 ± 53.28#(0.7)	306.62 ± 58.86^*^ (0.9)	281.5 ± 51.37#(2.0)	0.180	0.000	0.778	0.660
V_Emax_ (l·min^−1^)	141.12 ± 32.24	146.75 ± 34.58	150.37 ± 28.99	143.62 ± 23.46	0.785	0.525	0.919	0.105
V_Tmax_ (l)	3.06 ± 0.79	3.07 ± 0.72	3.04 ± 0.58	3.03 ± 0.65	0.779	0.911	0.628	0.865
BF_max_ (breaths·min^−1^)	57.25 ± 5.54	56.5 ± 7.72	57.12 ± 5.93	56 ± 7.38	0.468	0.711	0.488	0.850
**CG**								
VO_2max_ (ml·kg·min^−1^)	49 ± 8.37	43.78 ± 7.24	46.51 ± 4.1	42.67 ± 4.06#(2.3)	–	–	–	–
PPO (W)	306.62 ± 41.86	285.5 ± 43.1#(2.1)	307.75 ± 47.67	280.25 ± 44.65#(1.9)	–	–	–	–
V_Emax_ (l·min^−1^)	143.5 ± 35.97	148.5 ± 42.57	135.87 ± 40.92	147.12 ± 43.6	–	–	–	–
VT_max_ (l)	2.87 ± 0.75	2.93 ± 0.77	2.88 ± 0.8	2.87 ± 0.82	–	–	–	–
BF_max_ (breaths·min^−1^)	59.25 ± 10.87	62 ± 9.81	57.75 ± 14	59.12 ± 11.24	–	–	–	–

Data are presented as Mean ± SD and Effect size (ES). ES is showed when a statistical difference was found. VO_2max_, Maximum oxygen uptake; PPO, peak power output; V_Emax_, maximum ventilation; VT_max_, maximum tidal volume; BF_max_, maximum breathing frequency. ANOVA mixed-effects Bonferroni post hoc test:

* *Mixed-effects ANOVA Pre vs. Post in the same condition (p < 0.05)*.

# *Mixed-effects ANOVA Nor vs. Hyp at the same time (p < 0.05)*.

**Table 6:** **Correlation analysis between performance variables and ventilatory efficiency variables after experimental protocol with data from both groups**.

	**Pearson-r**				
	**W_TTmean_ (W)**	**VO_2max_ (ml·kg·min^−1^)**	**V_Emax_ (l·min^−1^)**	**Pi_max_ (cmH_2_O)**	**PPO (W)**
**NORMOXIA**					
Pimax_(cmH_2_O)_	0.607^*^	0.503^*^	0.859^*^	1	0.623^*^
V_E_/VCO_2_ slope	0.126	0.153	0.278	0.361	0.036
LEqCO_2_	0.011	0.083	0.288	0.274	−0.064
EqCO_2_VT_2_	−0.1	0.026	0.062	0.196	−0.220
OUES	0.89^*^	0.683^*^	0.669^*^	0.454	0.913^*^
**HYPOXIA**					
Pimax (cmH_2_O)	0.599^*^	0.477	0.545^*^	1	0.587^*^
V_E_/VCO_2_ slope	0.060	0.045	0.145	0.304	0.029
LEqCO_2_	−0.250	−0.084	−0.029	0.069	−0.283
EqCO_2_VT_2_	−0.105	−0.016	−0.019	0.131	−0.166
OUES	0.828^*^	0.664^*^	0.79^*^	0.408	0.885^*^

*Pimax, Peak inspiratory pressure; V_E_/VCO_2_ slope, Slope of the relationship between VCO_2_ and V_E_; LEqCO_2_, lowest equivalent of CO_2_ during the incremental test; EqCO_2_VT_2_, equivalent of CO_2_ in the second ventilatory threshold; OUES, oxygen uptake efficiency slope; VO_2max_, Maximum oxygen uptake; PPO, peak power output; VEmax, maximum ventilation; WTTmean, mean watts*.

* *Significant correlation (p < 0.05)*.

Some limitations have to be addressed. First, the sample size was large enough to detect changes in Pimax and performance within the intervention group but might have been too low to detect group differences. Second, we do not have exact data on the amount of training and competitions completed by the subjects apart from the inspiratory training load. However, participants were advised not to change their usual training habits during the experimental period. All participants were enrolled in the same practical courses, and all reported to only have limited time for sports outside of the university setting. In addition, it was reported that conventional training (no specific breathing training) does not improve breathing muscle strength (Illi et al., 2012). Therefore, we can assume that they completed approximately the same training apart from the IMT and this did not influence our results. Third, we did not measure the oxygen saturation (SaO_2_) during the hypoxia trials and this may have contributed important information. Lastly, we did not control for a possible placebo effect. However, as it is stated in the methods section, a large placebo effect is not expected. Therefore, we are confident that our conclusions were not affected.

## Conclusions

Even though sample size might have been too low to show an interaction effect, the results of the present study suggest a possible positive effect of IMT on cycling time trial performance in both normoxic and hypoxic conditions. Additionally, this study shows that hypoxia has a negative effect on the ventilatory efficiency and that IMT may reduce this effect. Finally, the data suggest that except or OUES, ventilatory efficiency measures seem not to affect cycling time trial performance. These findings may have relevance for athletes planning to complete a high altitude training camp or for athletes competing at high altitude. IMT before a competition at altitude might be a successful method to improve performance.

## Ethics statement

This study was carried out in accordance with the recommendations of Ethics Committee of the University of Innsbruck with written informed consent from all subjects. All subjects gave written informed consent in accordance with the Declaration of Helsinki. The protocol was approved by the Ethics Committee of the University of Innsbruck.

## Author contributions

Conception and design of the experiments: ES, AS, and JNO; Pre-testing, experimental preparation, data collection, and analysis: ES and HG. The first version of the manuscript was written by ES, HG, MB, JNO, and AS. All co-authors read and approved the final version of the manuscript.

### Conflict of interest statement

The authors declare that the research was conducted in the absence of any commercial or financial relationships that could be construed as a potential conflict of interest.
